# Exoscopic-assisted head and neck surgery: a comparative evaluation optimizing ergonomics and workflow

**DOI:** 10.1007/s11701-025-02551-7

**Published:** 2025-07-14

**Authors:** J. P. Lingl, T. K. Hoffmann, E. Goldberg-Bockhorn, J. Greve, F. Boehm

**Affiliations:** 1 Department of Otorhinolaryngology, Head and Neck Surgery, Ulm University Medical Center, Frauensteige 12, 89075 Ulm, Germany; 2 Surgical Oncology Ulm, i2SOUL Consortium, Ulm, Germany

**Keywords:** Exoscope, Exoscopic surgery, Head and neck surgery, Micro-surgery, Robotics, Ergonomics, Microvascular flap, Microvascular anastomoses, Cochlear implantation, Tympanoplasty, Parotidectomy, Otolaryngology, Robot-assisted microscopy, Surgical workflow optimization, Surgical education

## Abstract

Exoscopic surgery is an emerging visualization technique in head and neck surgery, offering a promising alternative to conventional surgical microscopes. AI-assisted functions and high-quality imaging support its integration into modern surgical workflows. Eleven head and neck procedures were evaluated, including otologic surgery, parotidectomy and microvascular anastomosis using exoscopic techniques. Subjective feedback was obtained from four primary surgeons and four surgical assistants (response rate 100%) via a custom, non-validated questionnaire comprising 18 items. Six questions compared exoscope versus standard magnification (microscope or surgical loupes) across domains including anatomical structure identification, depth perception, field illumination and magnification, preoperative setup handling, and overall preference. Twelve questions addressed ergonomic comfort, 3D glasses and head-mounted display (HMDmd) wearability, and intraoperative handling. Ergonomic strain was assessed using the validated Rapid Upper Limb Assessment (RULA). Exoscopic systems demonstrated advantages, such as superior 4K-3D imaging, enhanced depth perception, improved ergonomics and facilitated intraoperative collaboration and education. RULA scores indicated reduced musculoskeletal strain, especially during longer procedures. Mean setup time was 4:14 min (range: 4:05–4:45 min), including draping (mean 2:04 min; range: 1:50–2:30 min). Surgeons described a brief adaptation period, primarily related to foot pedal control and digital interface navigation. AI-assisted features, including Auto-Center and Voice Control, further optimized workflow. Exoscopic surgery offers benefits for head and neck procedures requiring sustained magnification and precision. Short training times and ongoing technological advancements, such as improved working distances, head-mounted displays, and cost efficiency, are expected to further enhance its clinical value.

**Supplementary Information:**

The online version contains supplementary material available at 10.1007/s11701-025-02551-7.

## Introduction

Microsurgical techniques have significantly enhanced the precision of surgical interventions in head and neck surgery. Since the introduction of the operating microscope in the 1950s, it has become an essential tool, particularly in microsurgical reconstruction, laryngeal procedures, and otologic surgery. [9] Over the past decade, the emergence of exoscopes has introduced a promising alternative to conventional optical systems. An exoscope traditionally consists of a camera suspended above a surgical field transferring images to a monitor. This allows the line of site to be independent of the surgical field as the eye is not tied to the ocular, as it is the case when using a microscope. Exoscopic surgery was first reported in the 1990s by Gildenberg, [7]. Initially used in neurosurgery, exoscopic techniques are increasingly being evaluated in (neuro) otologic surgery and head and neck reconstructive surgery [3].

Technological advancements have continuously enhanced the capabilities of the exoscopes, particularly through the integration of three-dimensional (3D) systems. Notable examples include the VITOM® 3D (Karl Storz, Tuttlingen, Germany), the ORBEYE^TM^ (Olympus, Tokyo, Japan) and the RoboticScope®-system (BHS Technologies®, Innsbruck, Austria). [1, 4] These state-of-the-art, digitally enhanced visualization systems provide high-resolution, 3D imaging with adjustable magnification allowing for greater flexibility in surgeon positioning and offering potential ergonomic advantages. However, these systems differ in their intended use and functionality: for instance, VITOM® 3D and ORBEYE^TM^ are fully exoscopic platforms, primarily relying on external camera units and monitors without integrated oculars. RoboticScope® features a head-mounted control interface with robotic arm guidance. A conventional direct view of the surgical field, as with a microscope, is not possible with this system either. Visualization is provided exclusively via a head-mounted display or a connected monitor.

In this article, we present our experience with an advanced hybrid surgical microscope, the KINEVO® 900 S from ZEISS, which uniquely combines both conventional microscopic and exoscopic visualization modes within a single device. In addition, it offers integrated robotic functionalities (e.g., Point-Lock, Z-Mode, Auto-Center) and AI-supported voice control, enabling precise and efficient workflow customization during surgery.

By analyzing a series of standard head and neck procedures performed with the ZEISS KINEVO 900 S, we evaluate the advantages and challenges of exoscopic surgery and assess its clinical applicability and potential future role in modern head and neck surgery. A systematic literature search was performed beforehand in PubMed using combinations of the terms “exoscope” with “head and neck”, “ear”, “cochlear implant”, “middle ear”, “parotid gland”, and “free flap”. All titles and abstracts were screened, and no prior publications were identified using the KINEVO® 900 S as an exoscopic device in this context. To the best of our knowledge, this is the first study to describe this new exoscopic system for use in head and neck surgery.

## Materials and methods

### Material

For this study, the ZEISS KINEVO 900 S surgical microscope was utilized, a state-of-the-art hybrid system that combines conventional optical microscopy with advanced robotic assistance, exoscopic capabilities, and AI integration.

**Fig. 1:**
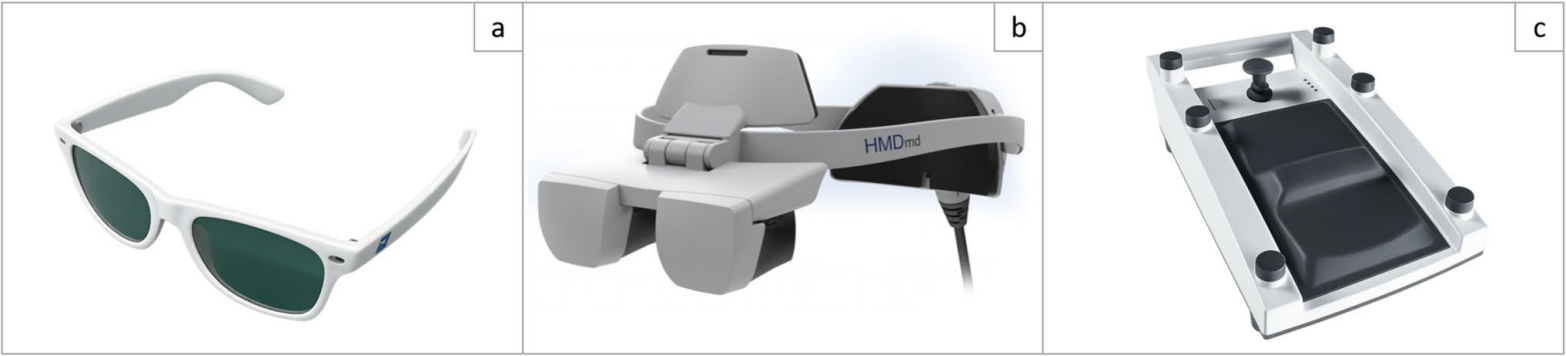
**a** Three-Dimensional (3D) glasses for the KINEVO® 900 S from ZEISS which allow for 3D-view on the system`s two monitors, **b** Head-mounted display (HMDmd) for the ZEISS KINEVO 900 S with visualization of the surgical field in 3D without the necessity of a direct view of the monitors for visualization, **c** Foot pedal for the ZEISS KINEVO 900 S with customizable buttons allowing for a hands-free control of the exoscope

The system uses native 4 K-3D visualization, providing high-resolution imaging with enhanced depth perception. Figure 1a shows the system’s 3D glasses. The system can be adapted to different surgical requirements: for a fully exoscopic approach, all ocular tubes can be removed, while for a hybrid setup, the microscope can be equipped with either a single or dual opposing ocular tubes, depending on the surgeon’s preference.

In addition, the system includes a head-mounted display (HMDmd), as shown in Fig. 1b, which is wired to the operating microscope and allows the surgical assistant to visualize the surgical field in 3D. The display can be dynamically adjusted to present a mirrored image of the surgeon’s view, ensuring correct left–right orientation from the assistant’s perspective and facilitating optimal spatial orientation.

The microscope is also equipped with a full range of standard optical filters to enhance tissue differentiation through fluorescence and contrast-enhanced imaging.

The exoscope can be controlled using standard handlebars or with a wireless foot pedal, allowing for hands-free operation. Some of the buttons can be customized to perform specific functions, improving workflow efficiency and adaptability to the individual preferences of the surgeon. Figure 1c depicts the foot pedal.

A key innovation of this system is the Cobotic Assistant Functionality, which integrate robotic support for advanced microscope movement.

- *XY-Mode*: Enables robotic-assisted translation of the microscope head along the same horizontal plane, allowing seamless adjustment of the field of view without manual repositioning.
- *Z-Mode*: Designed specifically for exoscopic surgery. This mode allows the microscope to move along the Z-axis (defined as the vertical axis above the surgical field). This feature permits adjustments to the working distance while maintaining a consistent viewing angle, eliminating the need for manual refocusing, as the system automatically maintains focus.
- *Point-Lock Mode*: This function enables the microscope head to move along a spherical trajectory around a fixed focal point, providing alternative viewing angles without losing focus on the target structure. This allows the surgeon to observe anatomical details from different perspectives while maintaining a stable field of view.
- *Position Memory*: The system allows surgeons to save specific microscope positions and focal points, enabling a quick return to previously defined perspectives during surgery.

ZEISS KINEVO 900 S is among the first surgical microscopes to integrate AI-assisted functionalities, further enhancing workflow efficiency:

- *Auto-Center*: This feature automatically detects and centers the instrument tip within the surgical field, ensuring that the visualization continuously follows the surgeon’s working area. The microscope can be realigned to the instrument’s position with a single press of the foot pedal or a hand switch command. This reduces the need for manual adjustments.
- *Voice Control*: The system includes voice recognition technology, enabling hands-free control of various functions, including initiation of photo and video documentation, adjustment of brightness and fluorescence modes and activation of the position memory function, which allows to store the microscope position.

### Methods

First, we conducted an inventory analysis of standard otolaryngologic procedures, that seem suitable for exoscopic surgery. Procedures that typically require optical magnification using loupes or a microscope were identified. Endoscopic procedures such as sinus surgery were excluded, because the endoscopic approach is the gold standard in these procedures and causes fewer complications than the microscopic approach. [10] Transoral tumor surgeries were also excluded from the study, as laser surgery is the standard of care in these cases, and no exoscope has yet been equipped with a laser. Special attention was given to musculoskeletal-straining procedures. A literature review was conducted to explore previous or potential new applications of exoscopes in head and neck surgery, focusing on reported advantages and disadvantages.

A standardized time measurement was conducted for the preparation of the exoscope, including startup time, sterile draping, and positioning of the microscope over the surgical site. If feasible, the entire surgical procedure was performed using only the exoscope. A change to conventional techniques (microscope or surgical loupes) was made, if necessary, in the form of a hybrid approach.

Throughout the procedures, the Cobotic Assistant Functionality was systematically tested and evaluated for feasibility and impact on the surgical workflow. The system was actively operated via both handlebars and the wireless foot pedal, which features customizable buttons to enhance workflow efficiency. The surgeons were encouraged to use the wireless foot pedal exclusively in the beginning of the surgery for testing, later they could switch to the operating device they preferred. A full range of optical filters and standard visualization modes were available, though not specifically analyzed in this study. To familiarize themselves with the system, surgeons systematically tested all available robotic and AI-supported functions during their first procedures, including Voice Control and Auto-Center. These features were thereafter used at the surgeons’ discretion throughout the series, depending on procedural requirements and personal preferences. Both the 3D glasses (Fig. 1a) and the head-mounted display (HMDmd; Fig. 1b) were used for surgical visualization.

Following each procedure, subjective feedback was collected from the operating surgeons and surgical assistants.

Specifically, four experienced head and neck surgeons, two ENT-specialists, a resident and a final-year medical student completed structured custom evaluation questionnaires. The questionnaires were handed out immediately postoperatively, and the surgeons were asked to return them within three days. Responses were not anonymous, as each participant was part of the surgical study team and provided feedback openly in the context of internal performance evaluation.

The questionnaire was based on the previously published tools assessing exoscopic surgery in pediatric head and neck surgery by Chebib et al. (2023), European Archives of Oto-Rhino-Laryngology, doi: [2] and microneurosurgery by Takahashi et al. (2018), Clinical Neurology and Neurosurgery, doi: [11] From the Chebib et al. questionnaire, nine items were adopted without modification, except that the term “VITOM® 3D” was replaced with “KINEVO® 900 S.” From Takahashi et al., eight items were similarly adapted (replacing “ORBEYE™” with “KINEVO® 900 S”), and one additional custom question was added for this study to address the comfort of the head-mounted display.

The complete adapted questionnaire is provided as Supplementary Material.

Ergonomic assessment was performed using the Rapid Upper Limb Assessment (RULA) method [8] to evaluate musculoskeletal strain associated with exoscopic surgery compared to conventional procedures using surgical loupes or microscopes. RULA is a validated tool to assess upper limb, neck, and trunk strain, assigning scores based on joint angles and muscle activity. Higher scores indicate a greater need for ergonomic intervention. The RULA Assessment was completed by the participants (surgeons and assisting physicians) themselves, with the data collection form being handed immediately postoperatively. The surgeons were asked to return the forms within three days.

## Results

### Analysis of applications suitable for exoscopic surgery

The analysis identified the most suitable surgical applications for exoscopic surgery within head and neck procedures, specifically for otologic, oncologic, and cervical soft tissue surgeries. From an ergonomic perspective, exoscopic surgery could provide a significant advantage for these types of procedures, where surgeons and assistants often find themselves maintaining a flexed neck posture during traditional surgeries.

Exosopic surgery was found to be particularly beneficial in parotid surgery, where enhanced visualization can improve surgical precision for both the primary surgeon and the assisting surgeon. A major risk in parotid gland surgery is injury to the facial nerve, so these procedures are routinely performed with neurophysiologic monitoring and direct optical assessment by an assisting surgeon at our institution. The assistant typically stands opposite the primary surgeon and observes the patient’s sterilely draped face for any involuntary movements, indicating nerve stimulation. However, this position offers limited direct visualization of the surgical site. Exoscopic visualization, displayed on multiple monitors or through the HMDmd, could enhance the assistant’s view of the surgical field, possibly allowing for better intraoperative participation and improved surgical training opportunities.

Another promising application is microvascular soft tissue surgery, particularly for anastomotic suturing in free flap reconstruction. Most commercially available surgical microscopes are designed primarily for neurosurgery, hand surgery, ophthalmology, or otologic surgery, where the line of sight is either directly overhead (as in neurosurgery and ophthalmology) or laterally oriented for a single surgeon (as in otologic procedures). However, microvascular anastomoses in cervical soft tissue reconstructions are often performed by two surgeons, an operator and an assistant, using dual opposing ocular tubes on the microscope. The main limitation in these cases is that the microscope is positioned obliquely lateral to the cervical soft tissues, and the ocular tube of the assistant sitting opposite of the surgeon is often too short for comfortable viewing and manipulation.

This limitation is addressed by the exoscope, which allows both the primary surgeon and assistant to view the surgical field simultaneously on high-resolution 3D monitors or through head-mounted displays. As a result, both team members can maintain ergonomic working positions independent of physical access to oculars, which enhances cooperation, precision, and overall surgical flow in microvascular procedures.

### Conduction of the surgeries with exoscopic approach

**Fig. 2:**
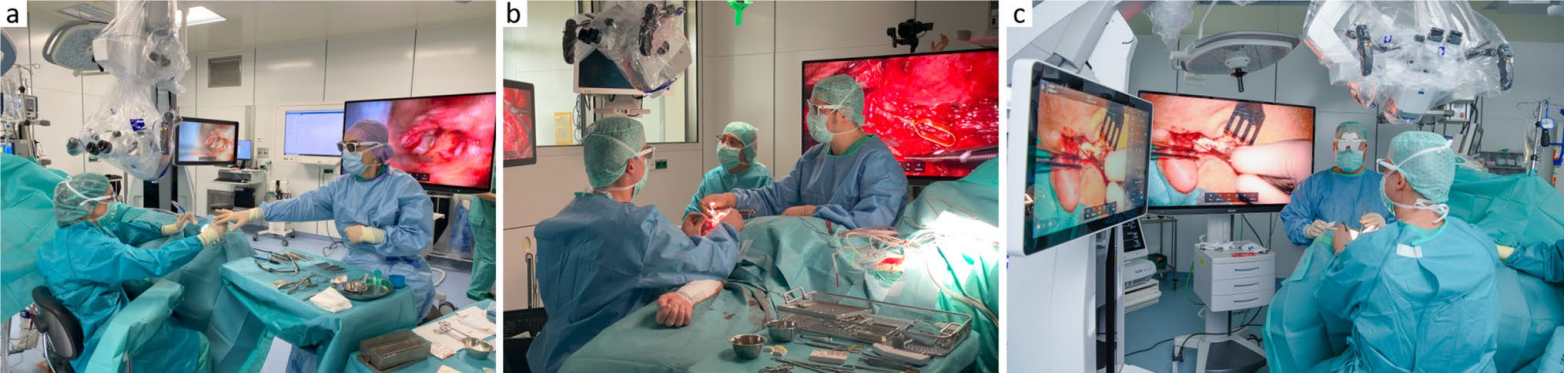
Operation room setup with placement of the KINEVO® 900 S from ZEISS. The ZEISS KINEVO 900S is seen in sterile draping, the oculars and the hand bars are still attached. The ZEISS KINEVO 900S is placed in adequate distance from the surgical field, without interfering with the surgeons’ movements or the surgical field. The figure shows exemplarily **a** a cochlear implant surgery, the surgeon on the left side is currently conduction mastoidectomy, both the surgeon and the assistant wear 3D-glasse, **b** the conduction of a microvascular anastomosis with the usage of the 3-D glasses for both surgeon and assistant currently conducting the venous anastomosis of a radial free flap and **c** a parotidectomy with the assisting surgeon using the Head-mounted display (HMDmd). The system is currently limited to the two presented monitors, the assisting surgeon is not looking at a third monitor, the visualization of the surgical field is possible with the HMDmd only

Following the identification of these surgical applications, a total of 11 exoscopic/hybrid procedures were conducted, consisting of three cochlear implantations, one intracochlear schwannoma resection followed by cochlear-implantation, four tympanoplasties (including two revision cases), two parotidectomies and one microvascular anastomosis of a radial free flap. The exoscope was used and evaluated by four experienced head and neck surgeons with assistance from two ENT-specialists, a resident and a final-year medical student. There were no complications during or after the surgery. Figure 2 shows the operation room setup, for cochlea implant (CI) surgery (a), for the microvascular anastomosis (b) and for the parotidectomy (c).

Nine out of these eleven cases were conducted entirely exoscopic. In one case, the second parotidectomy, we decided to start the surgery with the preparation of the skin flap and visualization of the parotid capsule without magnification, as we noticed a time and comfort disadvantage when starting exoscopic in the first parotid surgery (20 min vs. 30 min). In one of the three standard cochlear implant surgeries and during the resection of the intracochlear schwannoma, the surgeons opted to switch to the microscopic view for the insertion of the CI electrode and the wide cochleostomy respectively, as they felt more comfortable with the familiar technique and were still adapting to the new approach. The switch between exoscope and microscope was easy and seamless, thanks to the ZEISS KINEVO 900 S’s hybrid capabilities, which allow it to function as both an exoscope and a microscope.

An overview of the procedure types, number of cases, and surgical approach is provided in Table 1.

**Table 1:** Overview of procedures, number of cases, and surgical approach

Procedure type	Number of cases	Surgical approach
Cochlear implantation (CI)	3	2 fully exoscopic, 1 hybrid
Intracochlear schwannoma resection + CI	1	Hybrid
Tympanoplasty (incl. 2 revisions)	4	fully exoscopic
Parotidectomy	2	1 fully exoscopic, 1 hybrid
Microvascular anastomosis (radial free flap)	1	fully exoscopic

This table summarizes all procedures (*n* = 11) included in the study, listing the surgery type, the number of cases, and whether a fully exoscopic or hybrid (combined with conventional microscopy or surgical loupes) approach was used. Hybrid approaches were chosen in specific procedural phases based on surgical preference

No facial nerve injuries, wound healing disturbances, or exoscope-associated complications were observed. One instance of transient dizziness occurred after cochlear implantation, and one tympanoplasty required intraoperative prosthesis exchange due to anatomical complexity. In all other cases, postoperative outcomes were uneventful and consistent with the expected clinical course.

### Time measurement

The startup time for the ZEISS KINEVO 900 S was on average 1:38 min, which is highly efficient given the system’s complexity. The time required for sterile draping of the exoscope was 2:04 min (range 1:50–2:30 min), which is approximately the same as the time needed for draping a conventional microscope. The total setup process, including draping and positioning the microscope over the surgical site, took an average of 4:14 min (range 4:05–4:45 min). 

Mean time for cochlea implant surgery (incision-to-suture time) with the exoscope was 104 min. Depending on anatomic conditions cochlea implant surgery takes regularly between 80 and 150 min with a conventional microscope. Mean time for tympanoplasty was 102 min (conventional microscope 60–150 min), for the parotidectomy 135 min (conventional microscope: 60–180 min) and for microvascular suturing of the artery 19 min and of the vein 25 min (conventional microscope: ca. 20–30 min each for artery/vein).

### Ergonomic assessment

Ergonomic assessment was conducted in all eleven procedures for the primary surgeon and the assisting surgeon separately using the RULA method demonstrated superior ergonomics for all procedures performed compared to conventional techniques. This benefits both the primary surgeon and the assistant, as seen in Table 2.

**Table 2:**
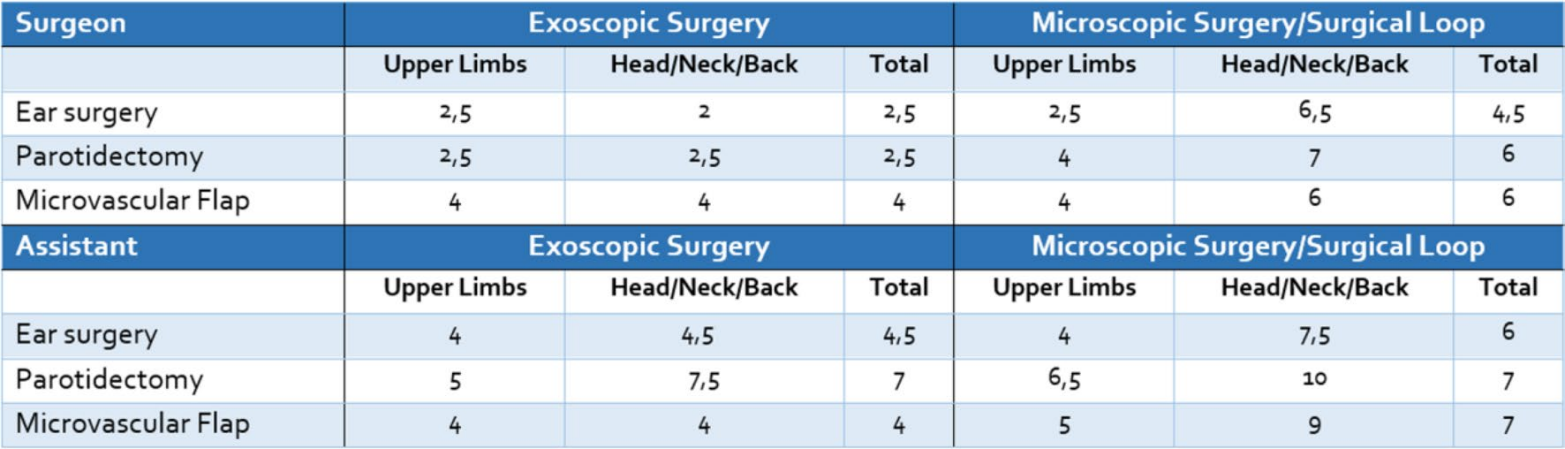
Average ergonomic stress according to the Rapid Upper Limb Assessment (RULA), a screening tool designed to quickly assess ergonomic risk factors that may contribute to musculoskeletal disorders (MSDs) of the upper limbs

It evaluates posture, movement, and biomechanical loads affecting the neck, trunk, and upper extremities during specific tasks. We conducted the RULA with separate assessment of the different procedures and divided between primary surgeon and surgical assistant. Ergonomic results were compared between exoscopic surgery and the standard technical device that is normally used during the procedure (microscope for ear surgery and microvascular flaps, surgical loupes for parotidectomy). The higher the RULA score the higher the ergonomic stress for the surgeon. A score 1–2 equals a low ergonomic negligible risk with no action required. 6 and more is a very high risk with immediate changes in ergonomics recommended

In cochlea implant surgery and tympanoplasty, the standard use of a microscope caused significant strain on the head and neck. One surgeon exhibited hyperextension of the neck and back to achieve the desired viewing angle, while another experienced up to a 20-degree neck flexion when using the microscope’s ocular. Forward bending of the upper body and neck was also observed during parotidectomy and microvascular anastomosis, especially for the assistant surgeon. These issues were largely addressed by the exoscopic approach, as it allowed for a straight posture of the head and back, regardless of the desired field of view.

In terms of ergonomic strain on arms and hands, all surgical procedures showed moderate upper limb strain. However, this could only be partially improved through the exoscopic approach.

### Visualization/evaluation questionnaire

**Fig. 3:**
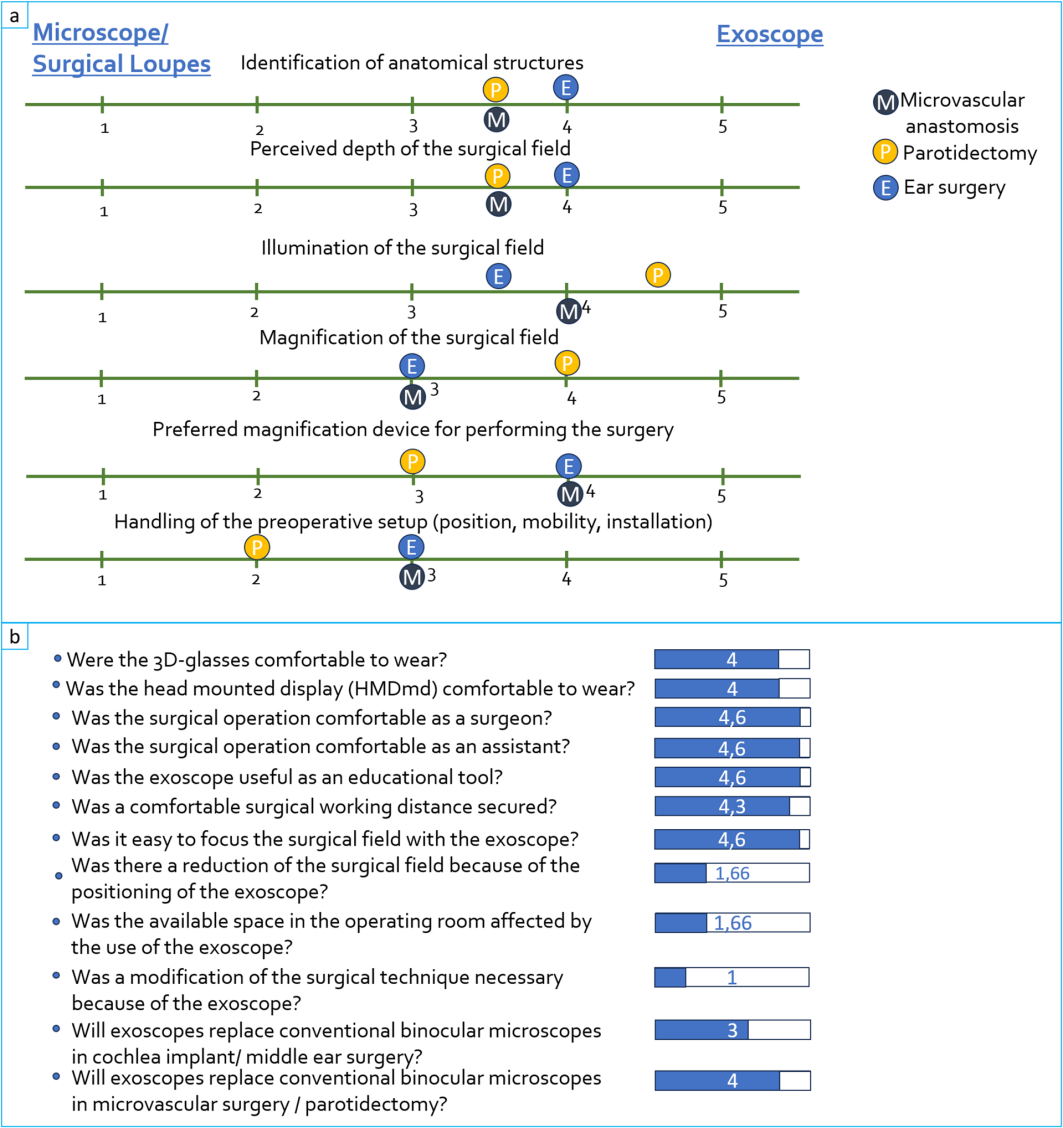
Surgeons’ evaluation (n = 8) of the KINEVO® 900 S according to a custom evaluation questionnaire handed to each surgeon after surgery. **a** Summary of the surgeons’ evaluation concerning the comparison of the ZEISS KINEVO 900 S with other standard magnification devices (binocular microscope and surgical loupes). The surgeons rated on a 5-point Likert-scale which device was favored depending on the conducted surgery depending on different rating categories like the depth of the surgical field or illumination. The assessment was conducted for each type of surgery separately, in case of different answers from the surgeons a median value was formed. M = microvascular anastomosis, P = parotidectomy, E = ear surgery. 1 = microscope/surgical loupes clearly favored, 2 = microscope/surgical loupes slightly better, 3 = neutral, 4 = exoscope slightly better, 5 = exoscope clearly favored, **b** Assessment of the pros and cons of the ZEISS KINEVO 900 S concerning comfort and feasibility rated on a 5-point Likert-scale. In case of different answers from the surgeons a median value was formed. 1 = very poor/strongly disagree, 2 = poor/disagree, 3 = neutral, 4 = good/agree, 5 = very good/strongly agree

Figure 3a summarizes the surgeons’ evaluation concerning the comparison of the ZEISS KINEVO 900 S with other standard magnification devices (binocular microscopes and surgical loupes). Figure 3b shows the surgeons’ assessment of the pros and cons of the ZEISS KINEVO 900 S concerning comfort and feasibility.

The surgeries were performed without any problems or technical failures. A comfortable surgical working distance was maintained and there was no visual reduction of the surgical field due to the positioning of the exoscope. There were no significant modifications to the surgical techniques, and the operating room space was easily managed since the ZEISS KINEVO 900 S is similar in size to a traditional microscope.

Only in case of the parotidectomy the surgical loupes were seen as equivalent to the exoscope as magnification device. This was because one the one hand, handling an analog magnification tool, such as surgical loupes, requires significantly less effort compared to operating the technically sophisticated exoscope. On the other hand the magnification of the surgical field was described as better with the exoscope. Additionally, from the assistant’s point of view, the use of the exoscope as a teaching tool, justifies the effort required for preoperative setup and intraoperative handling.

Additionally, while surgical loupes only need to be put on, the exoscope requires powering up, connecting, and draping, which can be time-consuming. This gave surgical loupes an advantage in terms of preoperative ease of handling technical equipment. When comparing otologic and microvascular surgeries, there was no preference in the preoperative setup as the ZEISS KINEVO 900 S required similar preparation as a standard operating microscope.

**Fig. 4:**
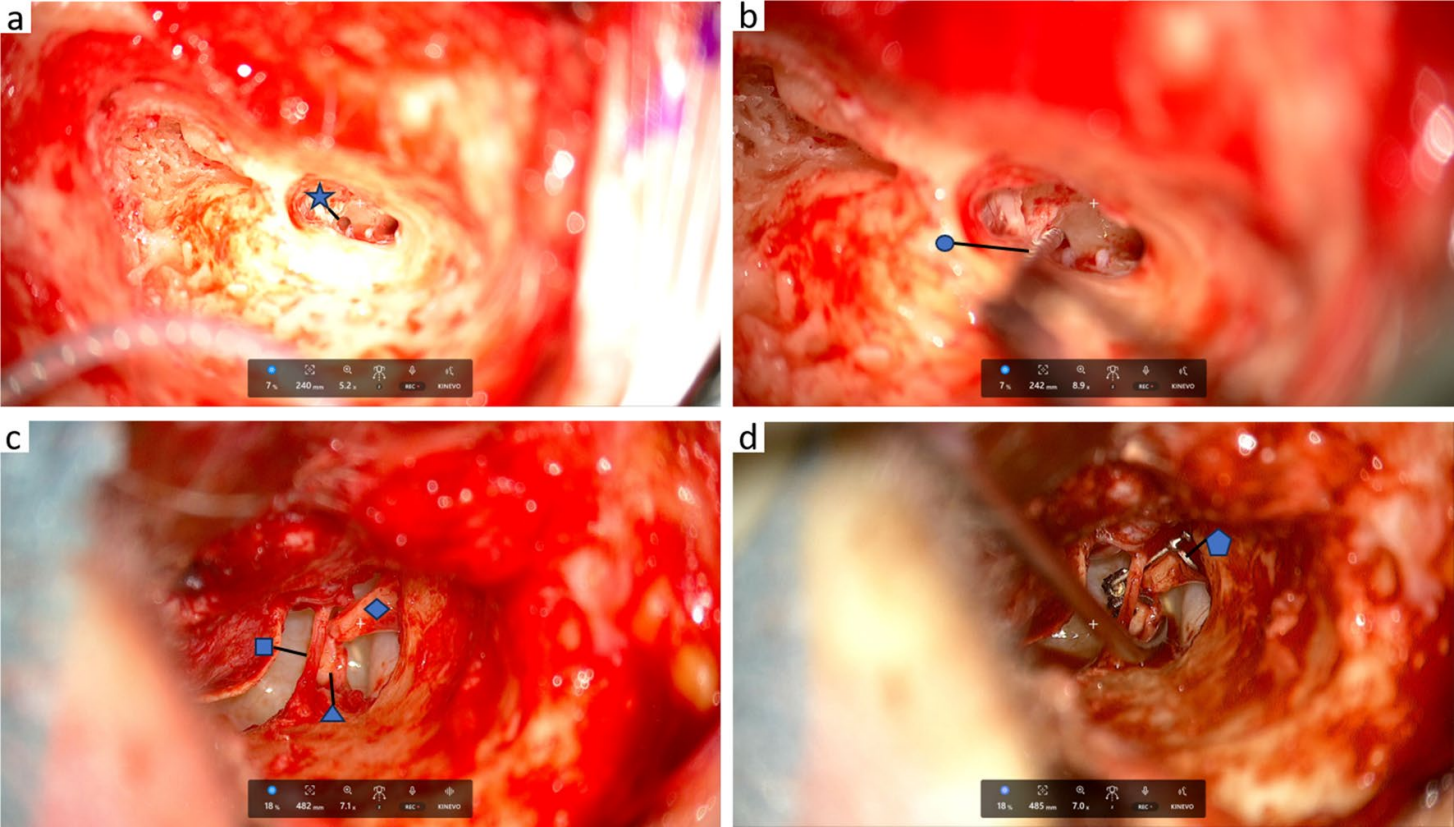
Intraoperative exoscopic image of the ZEISS KINEVO 900S in a case of cochlea implant surgery after mastoidectomy, antrostomy and cochleostomy (**a**) and insertion of the cochlear electrode into the round window (**b**), in tympanoplasty after mastoidectomy and antrostomy with visualization of the stapes suprastructure and the long process of the incus (**c**) and after placement of an angular clip protheses connecting the long process of the incus with the stapes suprastructure (**d**). round window visible after mastoidectomy, Cochlea Implant electrode inserted into the round window, chorda tympani, stapes superstructure, long process of the incus, angular clip prothesis connecting the long process of the incus and the stapes superstructure

**Fig. 5:**
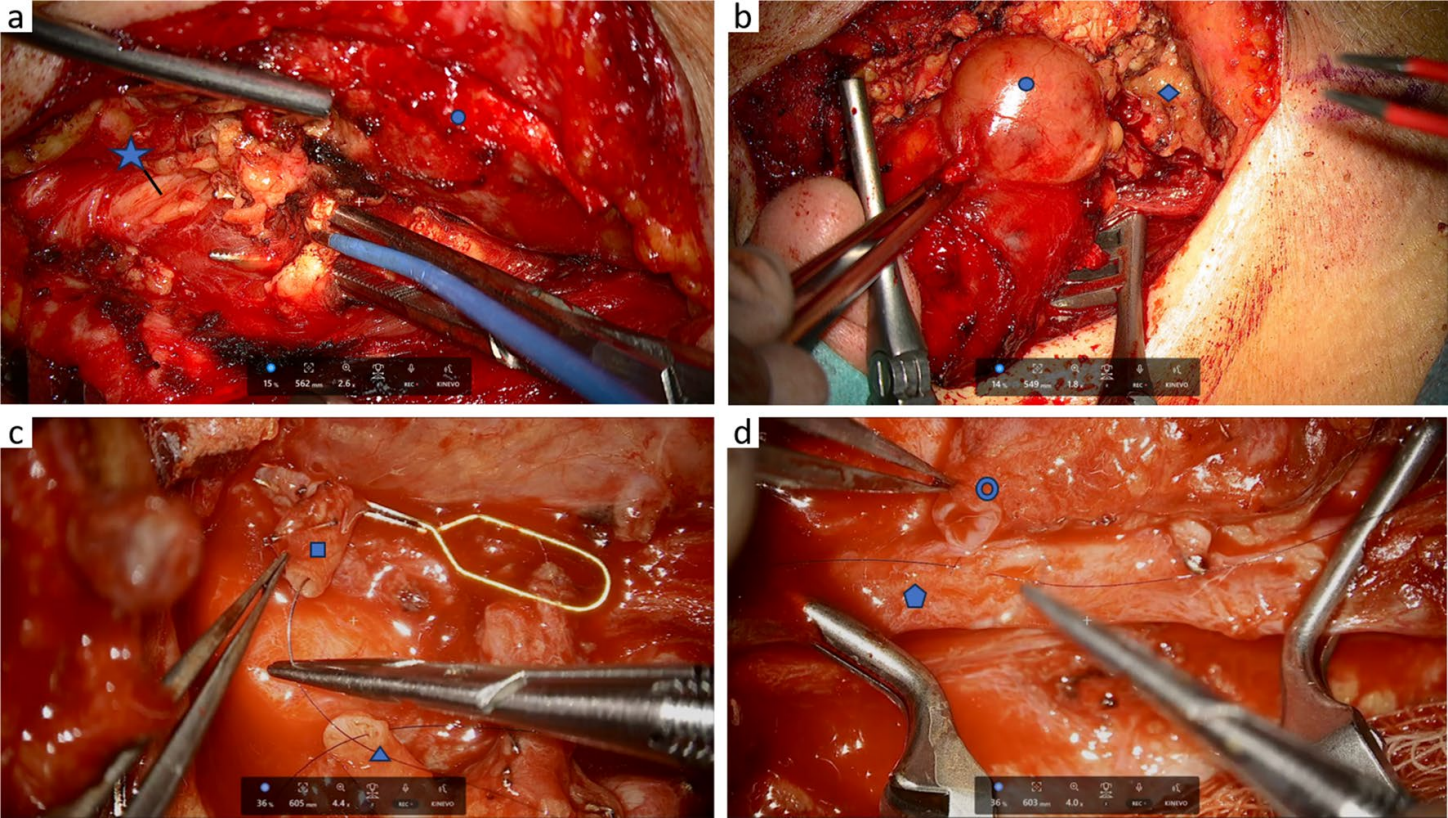
Intraoperative exoscopic image of the ZEISS KINEVO 900S during laterocaudal parotidectomy **a** with visualization of the facial nerve, the tumor mass is resected with the lateral part of the parotid gland, **b** resection of a parotid tumor mass. Intraoperative exocopic image during microvascular anastomosis of a radial free flap for reconstruction of an oropharyngeal defect after tumor resection (**c** + **d**) with arterial anastomosis of the radial artery and the superior thyroid artery (**c**) and venous anastomosis of the internal jugular vein and the median cubital vein (**d**). Facial nerve, tumor, healthy parotid tissue, Radial artery, superior thyroid artery, internal jugular vein, median cubital vein

The 4 K-3D resolution provided an exceptionally sharp and detailed visualization of the surgical field. The polarized glasses required for 3D viewing were comfortable to wear. They did not obstruct the view of the instruments or the surgical site when the head was turned away from the monitor. There have been no reported cases of headaches or fatigue during surgery due to wearing the 3D glasses. Overall, there was no noticeable difference in image quality between viewing the surgical field on the monitor with 3D glasses and through the oculars of the microscope. In middle ear surgery the depth of the surgical field and the identification of anatomical structures have even been described as being more favorable for the exoscope than the microscope. Figure 4 shows the intraoperative exoscopic image in a case of cochlear implant surgery (a + b) and in a case of tympanoplasty (c + d). During parotidectomy the illumination of the surgical field and its magnification were found to be better than with the use of surgical loupes. Figure 5 illustrates the intraoperative image during parotidectomy (a) with visualization of the facial nerve as well as the conduction of a venous and aterial anastomosis in the conduction of a radialis free flap (b).

All surgeons highlighted the educational benefits of the exoscopic view. Additional oculars mounted on conventional microscopes are often difficult to reach for the assisting surgeon or student. In addition, the orientation and the field of view through the assistant’s ocular is often slightly different from the surgeon’s view. Traditional microscope monitors have historically displayed a 2D image with less detail and brightness than the original image through the ocular. This was a disadvantage especially in middle and inner ear surgery, where orientation in a 2D image is difficult in the narrow and deep spaces of the middle and inner ear.

When testing the HMDmd, one of the assisting surgeons complained of headaches after approximately one hour of surgery due to the pressure and weight of the device. However, the visualization of the head-mounted display was described as accurate and well detailed. An advantage was that the surroundings in the operating room could be seen while wearing the head-mounted display. This is important for patient safety, especially during parotidectomy when facial nerve stimulation must be visually recognized.

### Robotic control and AI-assisted functionalities

One of the most significant adjustments for the surgeon involved adapting to microscope positioning via foot pedal rather than the conventional handgrip controls. While this required an initial adaptation period, it ultimately facilitated continuous bimanual operation and improved surgical efficiency. The foot pedal can be individually programmed for each surgeon, allowing for customization of robotic motion speed and control settings of the Cobotic Assistant Functionality.

The integration of Voice Control was particularly appreciated by the surgeons as it allowed hands-free operation of key functions, such as photo and video documentation, light intensity adjustment, position memory activation, and setting of different movement modes. This feature reduced the workload for the operating room staff and facilitated the control of the exoscope for the surgeon.

The Point-Lock function was particularly useful during otologic procedures such as tympanoplasty, allowing efficient inspection of the tympanic cavity from various angles without the need for repositioning. The ability to maneuver the microscope around a fixed focal point was also advantageous during parotid surgery and microvascular anastomoses.

The Z-mode facilitated smooth exoscopic operation but revealed its limitations when the system’s maximum working distance of 625 mm was reached. Autofocus performance was occasionally suboptimal at this range, requiring manual adjustments via foot pedal. In a smaller distance (up to 550 mm) the autofocus worked reliably.

## Discussion

The introduction of exoscopic surgery represents a significant advancement in head and neck surgery. Our findings highlight its distinct advantages in ergonomics, visualization, and surgical workflow. While transitioning from conventional microscopy requires an initial adaptation period, exoscopic techniques integrate well into otolaryngologic procedures, facilitated by ENT surgeons’ prior experience with endoscopic screen-based visualization.

A key observation during the learning phase was the tendency to initially operate macroscopically rather than taking full advantage of the 3D exoscopic visualization on the monitor. However, surgeons quickly adapted, recognizing the significantly enhanced depth perception and image quality provided by the 4 K-3D system. The only two instances that required a transition to a hybrid approach—using the microscope ocular to insert a cochlear implant electrode insertion and for the wide cochleostomy during resection of an intracochlear schwannoma—were driven by surgeon preference rather than technical limitations.

In comparison to conventional surgical microscopes, the startup time of the exoscopic system was slightly longer due to its digital functionalities. However, as modern operating microscopes increasingly incorporate digital imaging and documentation features, this difference is minimal and does not significantly impact surgical workflow. Although fully analog microscopes are still in use today, they are expected to be phased out within the next decade as they are no longer being produced by leading manufacturers.

Surgery times were comparable to those of traditional procedures performed with either surgical loupes or standard operating microscopes. This is consistent with the existing literature, which has found no significant differences in operative times for cochlear implantation or microvascular anastomosis between exoscopic and microscopic approaches. [2, 5]

Subjective feedback from the surgeons reinforced the objective ergonomic advantages demonstrated by RULA analysis. For example, during mastoidectomy, surgeons maintained an upright seated posture with the exoscope, whereas conventional microscopy required a semi-reclined position. This improved working posture reduces musculoskeletal strain and enhances long-term surgical endurance. The ergonomic advantage was particularly noticeable for the assistant surgeon during parotid surgery and microvascular anastomosis, where maintaining an optimal view traditionally requires significant neck and upper body flexion, often resulting in discomfort. Recent studies focusing on purely exoscopic systems have also reported substantial ergonomic improvements [2, 4, 6].

Importantly, the exoscope also helps compensate for height differences between the primary surgeon and assistant, as visualization is no longer bound to fixed oculars. Instead, both team members can work from individualized ergonomic positions using shared 3D monitors or head-mounted displays, reducing strain and improving comfort across varying body sizes.

The surgeons described that the 4 K-3D resolution provided an exceptionally sharp and detailed visualization of the surgical field. Due to the optical zoom function, the resolution was not impaired even in the highest zooms ranges. This differs from other previously described exoscope systems, where one of the largest drawbacks of the systems was the impeded resolution in high zoom ranges due to the use of digital zoom instead of optical zoom. In middle and inner ear surgery, these systems were less suitable than the surgical microscope due to limitations in image quality at higher magnification levels [5, 6].

Autofocus performance was described as hugely advantageous by the surgeons, despite reaching occasionally suboptimal performance at the system’s maximum working distance of 625 mm, in these cases occasionally manual adjustments via foot pedal were required. In a smaller distance (up to 550 mm) the autofocus worked reliably. Although this did not significantly impact workflow, an increased working distance of 800–900 mm would be beneficial for enhanced versatility.

**Fig. 6:**
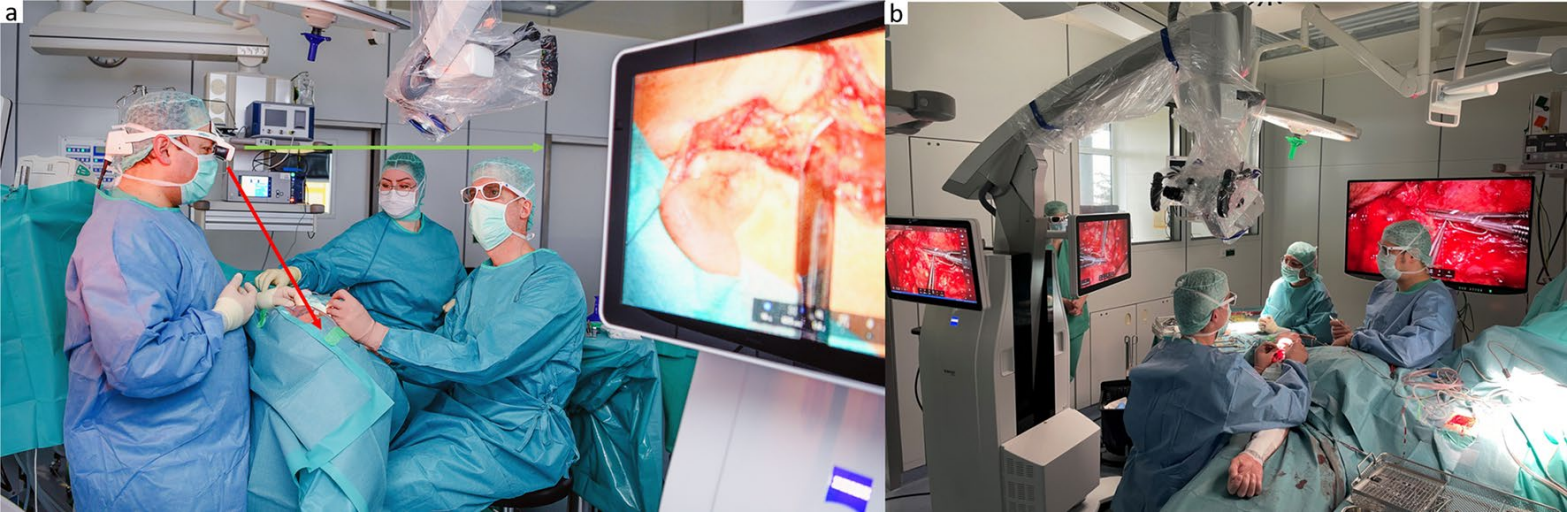
**a** Setup of the ZEISS KINEVO 900S during parotidectomy using the Head-Mounted Display (HMDmd). The green line illustrates the assistant’s direct line of sight to the surgical field via the HMDmd. The red line indicates the angle of neck anteflexion (approximately 30–40°) that would be required without the HMD to observe the surgical field without the exoscope. **b** Alternative setup using 3D glasses and external monitor (example from radial free flap surgery). This configuration is ergonomically acceptable but less favorable for parotidectomy, where continuous monitoring of facial movements is essential. The assistant must turn away from the patient’s face to view the monitor, limiting simultaneous nerve monitoring

The Head-Mounted Display (HMDmd) proved to be a game changer for surgical assistants, particularly in parotid surgery. For the first time, assistants had a direct insight into the surgical field while maintaining an unobstructed view of the patient’s face to monitor facial nerve reactions. A key advantage was that the monitor image could be digitally mirrored. This allowed the assistant to maintain their spatial orientation, even when positioned on the opposite side of the surgeon, without the need to adjust their perspective while assisting. Figure 6a illustrates this setup, including the assistant’s line of sight with and without the HMD, demonstrating how the device eliminates the need for uncomfortable neck anteflexion. As an alternative, 3D glasses and external monitors can also be used, as shown in Fig. 6b (depicted here during a radial free flap procedure). This configuration is ergonomically acceptable but less favorable for parotidectomy, where continuous monitoring of facial movements is essential. The assistant must turn away from the patient’s face to view the monitor, limiting simultaneous nerve monitoring.

The use of polarized 3D glasses for visualization was well received, but a better attachment solution is needed for surgeons who wear prescription glasses. In our study, adhesive fixation was used, but this is not a viable long-term solution.

No relevant image delay was noted by any of the users during surgery with the exoscope or when using the 3D visualization systems. Although latency-related discomfort is a known issue in virtual reality or 3D headset environments, none of the surgeons or assistants in our study reported symptoms of motion sickness, dizziness, or visual disorientation when using the 3D glasses. Mild headaches were reported after prolonged use of the head-mounted display (HMDmd), but these were attributed to its weight and pressure on the head rather than to optical or perceptual causes. Improvements in the comfort and design of the HMDmd are needed. Expected future developments include improved weight distribution by incorporating a head-supported design with a vertical support element, rather than relying solely on circumferential fixation. This approach helps distribute the load centrally on the head, similar to the design of a head mirror used in otolaryngology examinations. Currently, the HMDmd is only approved for use by assistants, but extending its certification to primary surgeons would be equally beneficial, as the surgeon would not have to look at a monitor at a certain distance. Surgical setup would also be simplified since the setup of the exoscope would not require as much emphasis on providing the surgeon with an unobstructed view to the monitor.

In parotid surgery, an efficient approach was to perform the skin incision, capsule preparation, and initial gland mobilization without magnification or with surgical loupes, switching to exoscopic visualization for dissection and facial nerve identification. This hybrid strategy optimized workflow, as early surgical steps do not require magnification. Attempts to perform these initial steps exoscopically led to frequent repositioning of the exoscope, unnecessarily interrupting the surgical flow.

Exoscopic visualization also enhanced teaching capabilities. The 3D monitor display provided superior visualization compared to traditional 2D surgical cameras, allowing observers to better appreciate anatomical structures and surgical maneuvers.

AI-assisted functionalities positively impacted surgical workflow. In particular, the Auto-Center function, simplified microscope positioning and reduced the need for manual adjustments, thus enhancing efficiency. The Voice Control enabled hands-free control of various functions, including initiation of photo and video documentation, adjustment of brightness and fluorescence modes and activation of the position memory function, allowing to store microscope positions. These functions were possible with the foot pedal as well, but the Voice Control was found to be more intuitive than the use of the foot pedal. In addition, the foot pedal has a limited number of buttons that can be assigned specific functions. Using Voice Control for a significant portion of the system’s operation, these buttons can be reserved for more specialized functions. In addition, with a little practice, the Voice Control will be much easier to handle than blindly using the foot pedal.

Although all procedures can be safely performed with traditional techniques, exoscopic surgery offers compelling advantages. In otologic surgery, it presents a particularly intriguing alternative to endoscopic methods. The Point-Lock feature, when combined with optimal patient positioning and an appropriate surgical approach—such as a sufficiently wide ear canal or upfront meatoplasty—allowed for visualization comparable to endoscopic techniques while maintaining the ability to perform bimanual surgery. This combination of enhanced visualization and preserved surgical flexibility makes exoscopic surgery an attractive furture-orientated technique.

The primary drawback of the ZEISS KINEVO 900 S is its high acquisition cost. However, when comparing this hybrid system to purely exoscopic competitors such as VITOM® 3D (Karl Storz, Tuttlingen, Germany) the ORBEYE (Olympus, Tokyo, Japan) and the RoboticScope®-system (BHS Technologies®, Innsbruck, Austria), the hybrid functionality of the ZEISS KINEVO 900 S offers a distinct economic advantage. Its ability to seamlessly switch between exoscopic and conventional microscopic views makes it a cost-effective option for institutions investing in high-end surgical microscopy.

This study has several limitations that must be considered when interpreting the results. First, the sample size is small (11 procedures), and all cases were conducted at a single tertiary care center by a limited number of experienced surgeons. Therefore, the generalizability of the findings to other institutions, surgical teams, or less experienced users may be limited. Second, the study was not randomized, and surgeon preference influenced the choice between exoscopic and hybrid techniques in individual steps. Finally, although subjective evaluations and ergonomic measurements provide valuable insight, further validation in larger, multicenter studies is necessary to confirm these results and better define the role of hybrid exoscopic systems in head and neck surgery.

Although the equipment was provided with support by the manufacturer (Carl Zeiss Meditec AG), no financial incentives were given, and the evaluation was conducted independently. Apart from a standard device introduction as required for the use of any new medical product, no further involvement by the manufacturer took place. All surgeries were performed autonomously by the treating surgeons.

## Conclusion

The introduction of exoscopic surgery represents a significant advancement in head and neck surgery. In this study, we evaluated the advantages and disadvantages of exoscopic surgery using a hybrid surgical microscope (ZEISS KINEVO 900 S) that integrates both conventional microscopic and exoscopic visualization. Our findings demonstrate that exoscopic techniques offer distinct advantages, particularly in otologic procedures, parotidectomy, and microvascular soft tissue reconstruction.

The system provided high-resolution 4 K-3D imaging, improved ergonomic working conditions, and seamless integration into surgical workflows. Objective ergonomic assessment using the Rapid Upper Limb Assessment (RULA) method confirmed a reduction in musculoskeletal strain when compared with conventional operating microscopes. In addition, exoscopic visualization enhanced intraoperative collaboration between the primary surgeon and assistant, while also improving surgical education through large-scale 3D visualization.

Despite these benefits, the transition to exoscopic techniques requires an initial learning curve, particularly in adapting to foot pedal controls, learning voice commands and leveraging the system’s digital functionalities. AI-assisted features, such as Auto-Center and Voice Control, proved to be valuable additions, optimizing workflow efficiency. 

Overall, our experience demonstrates that exoscopic surgery is a highly valuable addition to head and neck surgery. Despite an initial adaptation period, the ergonomic and visualization benefits, combined with AI-assisted workflow enhancements, make it a compelling alternative to traditional microscopic approaches. Future improvements in working distance, head-mounted display comfort, and cost efficiency will further strengthen its role in modern otolaryngologic surgery.

## Supplementary Information

Below is the link to the electronic supplementary material.

Supplementary file1 (PDF 190 KB)

## Data Availability

The data presented in this study are available upon request from the corresponding author.
