# Supplementary material for: Exoscopic-assisted head and neck surgery: a comparative evaluation optimizing ergonomics and workflow
Source: J Robot Surg. 2025 Jul 14;19(1):391. doi: 10.1007/s11701-025-02551-7 (PMC12259794; doi:10.1007/s11701-025-02551-7)
Supplement: Supplementary file 1 — Supplementary file1 (PDF 190 KB) [file 11701_2025_2551_MOESM1_ESM.pdf]

Evaluation Questionnaire Exoscope (Zeiss Kinevo 900s) vs. Standard Practice

Name of the surgeon:

Type of surgery conducted:

1) Compared to your usual practice (microscope or magnification surgical loupes) with the exoscope and concerning the **identification of anatomical structures/pathological tissues,** the performance of the exoscope was: (select one answer)
    - Much worse
    - Worse
    - Similar
    - Better
    - Much better
2) Compared to your usual practice (microscope or magnification surgical loupes), concerning the **depth of the surgical field,** the performance of the exoscope was: (select one answer)
    - Much worse
    - Worse
    - Similar
    - Better
    - Much better
3) Compared to your usual practice (microscope or magnification surgical loupes) concerning the **illumination of the surgical field,** the performance of the exoscope was: (select one answer)
    - Much worse
    - Worse
    - Similar
    - Better
    - Much better
4) Compared to your usual practice (microscope or magnification surgical loupes), concerning the **magnification of the surgical field,** the performance of the exoscope was: (select one answer)
    - Much worse
    - Worse
    - Similar
    - Better
    - Much better
5) Compared to your usual practice (microscope or magnification surgical loupes), concerning the **handling of the camera/device during the surgery,** the performance of the exoscope was: (select one answer)

- Much worse
- Worse
- Similar
- Better
- Much better

6) Compared to your usual practice (microscope or magnification surgical loupes), concerning the **preoperative setup** (position, mobility, installation of sterile fields) the performance of the exoscope was …: (select one answer)
    - Much worse
    - Worse
    - Similar
    - Better
    - Much better

Please answer the following questions using a 1-5 Scale, 0 for no answer:
5 very good/strongly agree
4 good /agree
3 neutral
2 poor/disagree
1 very poor/strongly disagree
0 not applicable / no answer

1) Were the 3D-glasses comfortable to wear?

2) Was the head mounted display (HMDmd) comfortable to wear?

3) Was the surgical operation comfortable as a surgeon?

4) Was the surgical operation comfortable as an assistant?

5) Was the exoscope useful as an educational tool?

6) Was a comfortable surgical working distance secured?

7) Was it easy to focus the surgical field with the exoscope?

8) Did the exoscope need more space in the operating room than your usual device (microscope/surgical loupes)?

9) Was there a reduction of the surgical field because of the positioning of the exoscope?

10) Was a modification of the surgical technique necessary because of the exoscope ?

11) Will exoscopes replace conventional binocular microscopes in cochlea implant/ middle ear surgery?

12) Will exoscopes replace conventional binocular microscopes in microvascular surgery / parotidectomy?
